# Combination of NK cells therapy and oral administration of entinostat as an approach for osteosarcoma lung metastasis treatment

**DOI:** 10.1186/2051-1426-3-S2-P25

**Published:** 2015-11-04

**Authors:** Simin Kiany, Ling Yu, Eugenie S Kleinerman

**Affiliations:** 1 University of Texas at Houston, MD Anderson Cancer Center, Houston, TX, USA

## Introduction

The goal of this study is to find an alternate therapy for osteosarcoma (OS) lung metastasis. Previously we showed that NK cell therapy significantly decreased OS lung metastasis in a mouse model; however, the therapy was not effective enough to cure OS lung metastasis. To augment the NK cell therapy, we elected to combine it with entinostat, an HDAC inhibitor. Studies have shown that HDACis sensitize tumor cells to NK cells cytotoxicity mostly by increasing expression of ligands for NK cells on tumor cells.

## Methods

Flow cytometry, western blot and Q-PCR were used to investigate whether entinostat increased expression of NK cell ligands on OS cells. Effects of entinostat on NK cell viability, receptor expression, and cytotoxicity were explored using, a viability test, flow cytometry, and calcein release assay, respectively. NK cells were isolated from blood buffy coats and were expanded *ex vivo* for 4 weeks using genetically engineered K562 cells and human IL-2. Q-PCR was used to measure microRNAs expression in OS cells. Ten, 5, or 2.5 mg/kg of entinostat were orally administered to mice to determine the sub-therapeutic dose of the drug for *in vivo* study.

## Results

We demonstrated that 2 μM entinostat for 48 h up-regulated expression of NK cell ligands (ULBP1, ULBP2/5/6, MIC A/B, and CD155) on OS cell lines (LM7, KRIB, CCHOSD, and CCHOSO). In addition, entinostat treatment increased susceptibility of OS cell lines to NK cell cytotoxicity. NK cell treatment with up to 2 μM entinostat did not neither affect the viability of NK cells nor expression of NK cell receptors (NKG2D, NKp30, NKp44, NKp46, and DNAM-1). NK cells pre-treatment with entinostat for 24 h did not decrease cytotoxicity of NK cell to OS cell lines. We also showed that entinostat increased MICA/B expression on OS cells by down-regulating miR-20a, miR-93, and mir-106b expression. We demonstrated that the sub-therapeutic dose of entinostat that significantly increased MICA/B on OS lung metastasis was 5mg/kg three times a week for 5 weeks.

## Conclusions

We demonstrated that entinostat immunosensitized OS cells to NK cell lysis by inducing up-regulation of ligands for NK cells on OS cells. Our results suggest that NK cell therapy in combination with entinostat is an innovative approach to enhance the immunotherapeutic effect of NK cells against osteosarcoma pulmonary metastases.

